# Bradyarrhythmia in COVID-19 Patients

**DOI:** 10.7759/cureus.19367

**Published:** 2021-11-08

**Authors:** Abdulrahman Hakami, Aisha Moafa, Abeer Alomaish, Maryam Mashraqi

**Affiliations:** 1 Internal Medicine, Jazan University, Jazan, SAU; 2 Medicine, Jazan University, Jazan, SAU

**Keywords:** coronavirus disease 19, severe acute respiratory syndrome coronavirus test, echocardiography, electrocardiography, computerized tomography, bradyarrhythmia, angiotensin-converting enzyme 2

## Abstract

The emergence of coronavirus disease 2019 (COVID-19) in 2019 has rapidly become a global health emergency. COVID-19 develops from a severe acute respiratory syndrome coronavirus (SARS-CoV) infection, which directly impacts the cardiovascular system by disrupting angiotensin-converting enzyme-2 receptors in the tissues. This leads to severe complications that cause major morbidity and mortality. Several cardiovascular complications have been reported during the pandemic, including myocardial infarction, stroke, pulmonary embolism, myocarditis, and tachyarrhythmias. Although bradyarrhythmia is another cardiac event associated with COVID-19, it has been reported in only a few cases in the medical literature. Here, we report two cases of young adult patients who were admitted because of a positive reverse transcriptase-polymerase chain reaction test of SARS-CoV-2 and presented with bradycardia detected on electrocardiogram but had an otherwise normal health condition with no history of cardiovascular illness.

## Introduction

Coronaviruses (CoVs) belong to the family Coronaviridae and carry a single-stranded, positive-sense RNA genome. The name “coronavirus” derives from the Latin and Spanish words for the crown “corona” to describe the crown-like spike proteins that appear on the surface of CoVs [1]. Severe acute respiratory syndrome coronavirus (SARS-CoV-2) are divided into four alpha (α), beta (β), gamma (γ), and delta (δ) variants. Among these, α and β are well-known to infect humans [2]. However, a vast outbreak of the δ variant was recently reported in India. Rapid mutation and recombination were also reported for CoVs. New cases of CoVs-related pneumonia reported in Wuhan, China, in November 2019, were later identified as β coronavirus, and the disease was named coronavirus disease 2019 (COVID-19) [3]. The World Health Organization (WHO) classified COVID-19 as a global pandemic on March 11, 2020. On January 1, 2020, Chinese scientists isolated severe acute respiratory syndrome coronavirus (SARS-CoV-2) from a patient and mapped the full genome sequence [4]. On February 11, 2020, the Coronavirus Study Group of the International Committee proposed the name SARS-CoV-2. Since its outbreak in Wuhan, the virus spread globally with almost 200 million cases and 4.1 million deaths as of July 2021 (WHO figures). Although continuing research efforts have provided a tremendous amount of information, many aspects of this unique coronavirus have yet to be elucidated.

Several studies have shown that COVID-19 is linked to cardiac symptoms, with a higher incidence in patients who were admitted to intensive care units. In the adult population, COVID-19 has been linked to several cardiovascular problems, the most common of which is acute myocarditis [5]. COVID-19 infection in young adult patients typically manifests with few or no symptoms but sometimes unusual or severe symptoms [6]. According to He et al., patients with COVID-19 had cardiovascular problems, including venous thromboembolism, atrioventricular (AV) block, elevated pulmonary artery pressure, and ST-segment elevation with multifocal ventricular tachycardia and a high cardiac troponin I (cTnI) level [7]. Infections have a significant effect on the cardiovascular system, according to clinical findings of illness patterns. In several situations, a history of cardiovascular illness impacts the severity of COVID-19 infections and contributes to clinical consequences. Viral infection induces cardiomyocyte damage through direct injury and secondary immune reactions, leading to myocarditis and dilated cardiomyopathy, but unclear mechanism with COVID 19 [5]. Furthermore, some medications used for COVID-19 infection like the combination of hydroxychloroquine and azithromycin, increased the risk for arrhythmias [7-8]. The mechanism underlying the pathogenesis of COVID-19 is that the virus binds tightly to soluble and cell-associated angiotensin-converting enzyme (ACE) 2 receptors, which are present in most organs, including the heart and lungs [9]. Patients infected with COVID-19 present with a spectrum of atypical cardiac manifestations and acute respiratory symptoms [10], whereas hospitalized patients are characterized by obvious respiratory symptoms, with respiratory arrest as the leading cause of death. However, the exact involvement of COVID-19 for cardiovascular manifestations is still in need of more extensive studies.

## Case presentation

Methodology

This is a retrospective case series of two patients with polymerase chain reaction (PCR)-confirmed coronavirus 2 (SARS-COV-2) infection who were admitted to our hospital in the southern region of Saudi Arabia from July 2020 to August 2020.

Following the admission of the patients, a careful examination of general parameters, such as temperature, pulse rate, consciousness, and reflexes response, was performed and recorded. After the general examination, blood was drawn from the patient for a detailed analysis of blood parameters utilizing a blood test, chest X-ray, electrocardiogram (EKG), transthoracic echocardiogram (Echo), and computed tomography (CT) scan to check the severity of the disease.

Case 1

**Figure 1:**
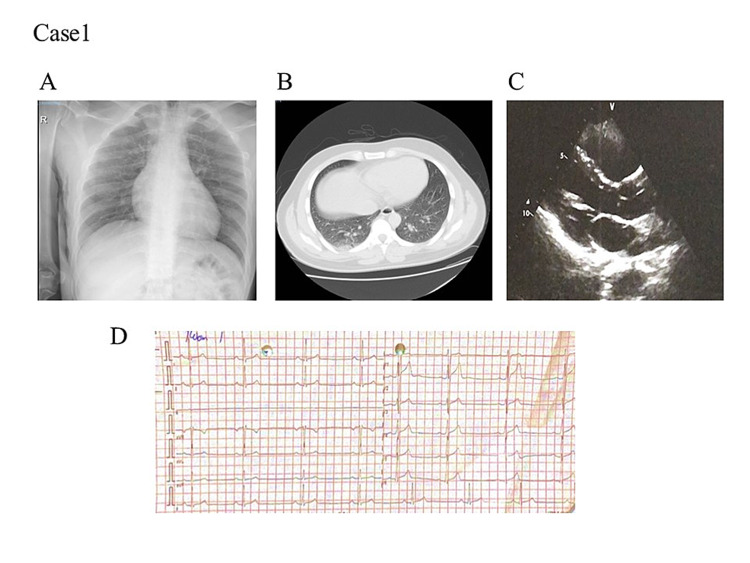
Medical examination of a patient (Patient 1) suffering from COVID-19 (A) Chest X-rays showing the normal view. (B) Chest computed tomography (CT) revealed a ground-glass appearance in the right lower lobe. (C) Echocardiography revealed a normal echo study. (D) Electrocardiogram (EKG) at admission showed prolonged QT interval and bigeminy.

A 34-year-old man with no previous history of chronic illness and a non-smoker presented with a history of headache, fatigue, diarrhea, vomiting, and insomnia for three days. During the initial examination, he was conscious and alert. His blood pressure (BP) was 111/71, pulse rate (PR) 40, respiration rate (RR) 14/min, body temperature 36.7, and oxygen saturation (SpO2) 96% under ambient oxygen conditions. The patient had a clear chest, without any crepitating sounds in the cardiovascular system (CVS; S1+S2+0). An abdominal exam showed a soft and lax abdomen, and both lower limbs were normal. The status of the central nervous system (CNS) was normal, all cranial nerves were intact, and chest X-ray and chest CT scans were performed (Figures 1A-1B). EKG showed sinus rhythm, first-degree heart block with prolonged QT interval, and bigeminy (Figure 1D). Echo revealed a normal echo study (Figure 1C). General clinical and blood parameters of the patients are shown in Table 1. Due to the COVID-19 pandemic, all patients reporting to the hospital with fever were routinely tested with the PCR test for COVID-19. Also, a nasopharyngeal swab was tested by RT-PCR and proved to be positive for SARS-CoV-2.

Case 2

Another 34-year-old male patient who smoked visited the hospital with a history of fever, runny nose, and diarrhea for seven days. There was no complaint of shortness of breath or chest pains. The patient was tested for central nervous system (CNS) response and reflexes and was alert and conscious. Examination of clinical vitals parameters was performed and recorded as BP: 126/76; PR: 43; RR: 21/min; body temperature: 36.6°C; oxygen saturation (SpO2): 98%; CVS: S1+S2+0; chest bilateral vesicular breathing, and no lower limbs edema. EKG reports showed sinus bradycardia with a prolonged PR interval and QT interval; a U wave was observed in V1 (Figure 1D). Chest X-ray and chest CT showed unremarkable findings (Figures 1A-1B). Echocardiography also revealed a normal echo study (Figure 1C). General clinical and blood parameters of the patients are provided in Table 1.

**Table 1:** Diagnostic tests of patients with COVID-19 and cardiovascular involvement

Parameters	Patient 1	Patient 2
Age (years)	34	34
Blood pressure (mmHg)	117/75	126/76
Pulse rate	40	43
Respiration rate	14/min	21/min
Oxygen saturation (%)	96	98
Sodium (Na) (mmol/L)	137	135
Potassium (K) (mmol/L)	4.3	4.4
Prothrombin time (PT)	12.8 sec	11.8 sec
Hemoglobin (Hb)	15.4	13.3
Activated partial thromboplastin time (APTT)	34 sec	38.7 sec
Lactate dehydrogenase (LDH) U/L	211	130
D-dimer ug/ml	0.88	0.27
Glycated hemoglobin (HbA1C) % (mmol/mol)	5.7	6.3
Ferritin ng/ml	295.8	157
Creatinine (0.6 to 1.3 mg/dL)	75	53
Creatine kinase (CK) U/L	52	39
Creatine kinase-MB (CK-MB) U/L	10	18
White blood cells (x10^9^/L)	4.05	7.25
Neutrophile cells (x10^9^/L)	1.65	1.98
Monocytes (x10^9^/L)	0.63	0.54
Lymphocyte cells (x10^9^/L)	1.69	4.25
Platelets (PLT) (x10^9^/L)	130	286
Alkaline phosphatase (ALP) U/L	64	58
Alanine transaminase (ALT)	83	18
Aspartate aminotransferase (AST)	35	17
Albumin (ALB) U/L	41.1	36.1
Gamma-glutamyl transferase (GGT) U/L	64	70
C-reactive protein (CRP) (0.3-10 mg/L)	24	5

Patient 2 was suspected of COVID-19 due to a non-symptomatic fever. The nasal swab of Patient 2 was tested by the same procedure as for Patient 1 and was SARS-CoV-2 positive.

**Figure 2:**
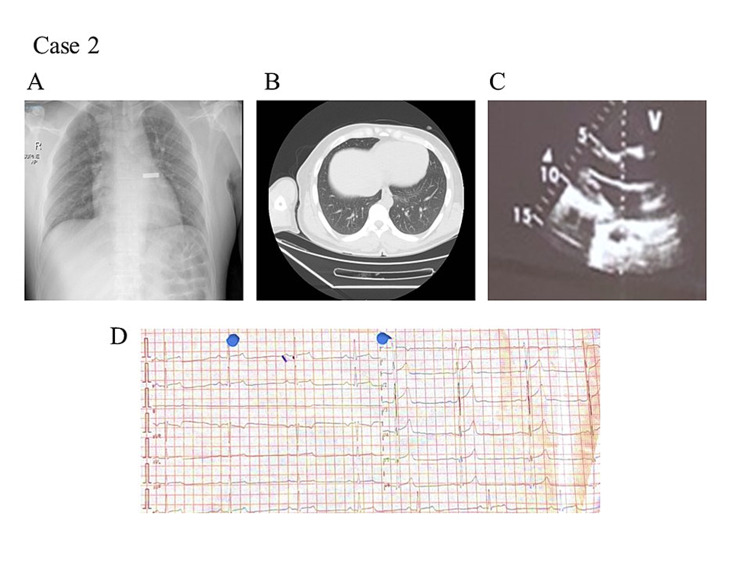
Medical examination of a patient (Patient 2) suffering from COVID-19 (A) Chest X-rays of the patient revealed no clear opacifications. (B) Chest computed tomography (CT) was normal. (C) Echocardiography showed a normal echo study. (D) Electrocardiogram recorded at admission showed decreased heart rate with prolonged PR interval and QT interval.

Treatment

Both patients were admitted to the isolation room and treated conservatively without hydroxychloroquine and azithromycin. They were administered 1 mg IV atropine and showed a transient change from bradyarrhythmia to sinus rhythm.

## Discussion

In most cases, a prior history of cardiovascular disorder impacts the severity of COVID-19 infections and leads to severe or fatal clinical consequences. Higher severity and mortality have been observed in patients with diabetes and hypertension and are associated with old age and male patients [11]. The early or mild phase of COVID-19 infection is typically characterized by "benign evolution" during the first seven days when the patient shows the typical symptoms of upper respiratory tract infection. Nonspecific symptoms, such as headache, sleeplessness, anosmia, and gastrointestinal manifestations, may also manifest as the disease progresses [12]. The patients at our hospital have had normal manifestations except for the early indication of a viral infection.

Furthermore, high levels of high-sensitivity troponin, C-reactive protein (CRP), ferritin, and leukocytosis were usually detected in severe acute cardiac damage and were closely related to cardiac arrest. These values demonstrate a high association with cardiac damage and hyperinflammatory response, which are attributable to viral infection [13]. In the present study, patients showed a higher than normal CRP. In a clinical case study, COVID-19 infection caused the up-regulation of CRP and creatinine kinase-MB (CK-MB) [14]. Myocarditis has been linked to viral infections on numerous occasions and has been recorded in patients with COVID-19 from the start of the current epidemic. In a case series of 150 COVID-19 patients conducted in Wuhan, China, 7% of 68 deaths (5 deaths) were related to myocarditis with circulatory failure; however, their pre-morbid cardiac state was unknown [15]. Previously, the incidence of COVID-19 myocarditis was reported in younger, healthy adults.

The data of previous case reports of patients infected with Middle East respiratory syndrome coronavirus (MERS-CoV) revealed elevated alanine transaminase (ALT) levels, aspartate aminotransferase (AST), and lactate dehydrogenase (LDH) due to liver function abnormalities [16]. Another study related to SARS patients' laboratory data showed high CRP levels, a decrease in lymphocytes, and high expression of aminotransferase, LDH, and creatine kinase [17]. In the present case study, patients' lymphocyte and platelets levels were low. However, the white blood cell count in Patient 1 was medium to high, and in Patient 2, it was high. Patient 2 showed more severe myocarditis and pulmonary edema due to a history of smoking, which can be a cofactor for COVID-19 severity. Arrhythmias are frequently the first symptom of myocarditis. Myocarditis, both acute and chronic, is one of the most common causes of progressive atrioventricular (AV) block in young and middle-aged patients. Almost 18% of the 3,055 patients in the European Study of the Epidemiology and Treatment of Inflammatory Heart Disease had high-grade arrhythmias, including complete cardiac arrest. However, patients in the present study had no known underlying cardiac health issues. The laboratory data and EKG reports suspected COVID-19 infection. The new-onset hypoxia and CT characteristics were consistent with COVID-19, which necessitated ward isolation and a PCR test to confirm the diagnosis.

Cardiac arrhythmias were previously described in COVID-19 patients; however, the descriptions are generally vague. Cardiac arrhythmias were reported in 16.7% of 138 hospitalized Chinese patients, although most were admitted to critical care (44.4% vs. 6.9%). The particular form of arrhythmias was not documented or published. Sinus node dysfunction has been documented in two isolated COVID-19 cases, but a high-grade AV block has yet to be described. Previously, viral infections, such as influenza, SARS, MERS, and parvovirus B-19, were the most common infectious cause of acute myocarditis. It is crucial to determine the different aspects of myocardial injury in patients with COVID-19. History of myocardial injury increases the severity and mortality of COVID-19 [18]. However, besides EKG and chest CT reports, blood parameters, including CRP, LDH, AST, ALT, and blood cell count, can be used to predict the onset of COVID-19 [18-19].

Sinus bradycardia reported in other viral infections includes viral hemorrhagic fevers, dengue fever, legionella, and malaria [20].

In our retrospective case series, the two COVID-19 patients presented early with bradycardia, which was not related to medications and was transient and reversed with atropine. Echo studies exclude abnormalities, which provides no clue for an unknown mechanism for bradyarrhythmia in COVID-19.

## Conclusions

In conclusion, bradyarrhythmia is an uncommon cardiac event in COVID-19. It might be transient but may lead to cardiovascular complications during the course of COVID-19. Therefore, clinicians must be aware of heart rhythm disorders that develop in COVID 19 patients. Prompt monitoring with cardiac evaluation is also needed. Further studies are warranted to specify the underlying pathogenetic mechanisms.
